# Effect of population stratification on the identification of significant single-nucleotide polymorphisms in genome-wide association studies

**DOI:** 10.1186/1753-6561-3-s7-s13

**Published:** 2009-12-15

**Authors:** Sara M Sarasua, Julianne S Collins, Dhelia M Williamson, Glen A Satten, Andrew S Allen

**Affiliations:** 1 Department of Genetics and Biochemistry, Clemson University, 100 Jordan Hall, Clemson, South Carolina 29634-0318, USA; 2 JC Self Research Institute of Human Genetics, Greenwood Genetic Center, 113 Gregor Mendel Circle, Greenwood, South Carolina 29646, USA; 3 National Center for Chronic Disease Prevention and Health Promotion, Centers for Disease Control and Prevention, 4770 Buford Highway, Atlanta, Georgia 30341, USA; 4 Department of Biostatistics and Bioinformatics and Duke Clinical Research Institute, Duke University, 2400 Pratt Street, Durham, North Carolina 27705, USA

## Abstract

The North American Rheumatoid Arthritis Consortium case-control study collected case participants across the United States and control participants from New York. More than 500,000 single-nucleotide polymorphisms (SNPs) were genotyped in the sample of 2000 cases and controls. Careful adjustment for the confounding effect of population stratification must be conducted when analyzing these data; the variance inflation factor (VIF) without adjustment is 1.44. In the primary analyses of these data, a clustering algorithm in the program PLINK was used to reduce the VIF to 1.14, after which genomic control was used to control residual confounding. Here we use stratification scores to achieve a unified and coherent control for confounding. We used the first 10 principal components, calculated genome-wide using a set of 81,500 loci that had been selected to have low pair-wise linkage disequilibrium, as risk factors in a logistic model to calculate the stratification score. We then divided the data into five strata based on quantiles of the stratification score. The VIF of these stratified data is 1.04, indicating substantial control of stratification. However, after control for stratification, we find that there are no significant loci associated with rheumatoid arthritis outside of the HLA region. In particular, we find no evidence for association of *TRAF1-C5 *with rheumatoid arthritis.

## Background

Population stratification occurs when a population is composed of subpopulations that have varying allele frequencies. When these subpopulations also have differing baseline risks for a trait, then population stratification can lead to spurious allele-trait associations. To control for confounding by population stratification in case-control studies, statistical methods have been developed that use genetic markers to provide information on population structure. Among such methods are genomic control [1,2], structured association [3,4], and principal components [5,6].

A new statistical approach for controlling for population stratification in case-control studies was recently proposed by Epstein et al. [7]. This method involves modeling the odds of disease, given data on substructure-informative loci. For each participant the stratification score, which is that participant's estimated odds of disease calculated using his or her substructure-informative-loci data, is calculated using the disease-odds model. Next, subjects are assigned to (typically five) strata defined by quantiles of the stratification score. Finally, the association between genotypes and the trait is ascertained using a stratified test. This approach is similar in spirit to the use of the propensity score to control for confounding in an observational study [8,9]. Epstein et al. showed that testing using the stratification score could control for confounding by population stratification in some situations where other methods fail [7].

The goal of this study was to assess the effect of controlling for population stratification in a genome-wide association study using the stratification score described above.

## Methods

We analyzed the genome-wide association study data from the North American Rheumatoid Arthritis Consortium (NARAC) provided as Problem 1 for Genetic Analysis Workshop 16 [10,11]. This dataset is composed of cases from several sources: families, sib-pairs, sporadic cases, persons with long time disease, and new onset cases. Control participants were selected from a population-based cancer study in New York, frequency-matched to case participants for self-reported ethnic origin. Genotyping was performed with the Illumina Infinium HumanHap550 (version 1.0) platform (San Diego, CA) with 545,080 single-nucleotide polymorphisms (SNPs) for all case participants and 48% of control participants; 33% of controls were genotyped using HumanHap550 version 3.0 and 20% with the HumanHap300 and HumanHap240S arrays. The multiple sources of case and control participants in these data argues for careful examination of the role of population stratification in any associations found.

We followed the basic quality control procedures outlined by Fellay et al. [12], excluding data from SNPs that had extensive missingness (missingness > 5%), deviations from Hardy-Weinberg equilibrium (*p*-value < 0.001 in controls), and low minor allele frequency (<1%). After removing duplicated and contaminated samples, information was available for 2058 individuals (868 cases; 1190 controls). Of these, 568 individuals were male and 1490 were female. A total of 501,228 SNPs were used in subsequent analyses. The average genotyping rate for subjects was 0.994. PLINK [13] was used for data cleaning and to calculate both the unstratified and stratified Mantel-Haenszel allelic association test. *p*-Values of the max(T) were computed using both the Bonferroni method and 10,000 permutation datasets.

We used the stratification score of Epstein et al. to adjust our analyses for confounding due to population stratification [7]. The authors focus on adjusting association tests using a limited number of ancestry-informative markers and, therefore, partial least squares (PLS) was used to estimate the stratification score. Here, no such marker panel was readily available; hence, we utilized markers from across the genome. Applying PLS to these data would likely result in substantial overfitting of the stratification score, leading to a loss of power [14,15]. In order to appropriately use this genome scale information, a different approach was needed. Thus we used a modified principal-component (PC) approach based on Fellay et al. [12] in place of PLS. Starting with the 501,228 SNPs that passed our quality control procedure, this modified PC approach captures the large-scale genetic variation in the data while minimizing the influence of a few regions high in linkage disequilibrium (LD) from dominating the PCs. This is accomplished by excluding SNPs from the PC analysis that reside in regions of known high LD and then further pruning the PC SNP set to minimize the LD between the remaining SNPs. After this pruning procedure 81,500 SNPs remained. Using the first few PCs, four individuals (D0009459, D0011466, D0012257, and D0012446) were found to be significant outliers, suggesting appreciable non-European ancestry. These individuals were excluded from subsequent analyses and, when the PC analysis was repeated, no further outliers were identified. The first 10 PCs were then used in a logistic model of disease to estimate each individual's stratification score--their predicted probability of being a case given the genomic information contained in their PCs. Five strata were then formed based on the quantiles of the stratification scores, for use in a stratified association analysis. We note that the computation demands presented by this procedure are quite minimal; it took approximately 30 minutes to generate the principal components and calculate the stratification score using a Linux workstation with two dual core 2.39-GHz opteron processors and 6 GB of RAM.

We measured confounding by population stratification using the variance inflation factor (VIF), defined as the median of the observed χ^2 ^test statistics divided by the expected value of this median under the null hypothesis of no association of any SNP with rheumatoid arthritis (RA) [1].

## Results

The unstratified analysis has a VIF of 1.44, while the VIF of the stratified analysis using the method of Epstein et al. was 1.034. In this context, it is worth noting that the identity-by-state (IBS) clustering approach to controlling for confounding by population stratification that is implemented in PLINK, and that was used by Plenge et al. [11], only attained a VIF of 1.14. For this reason, Plenge et al. also used genomic control [1,2] to control the residual confounding.

**Figure 1:**
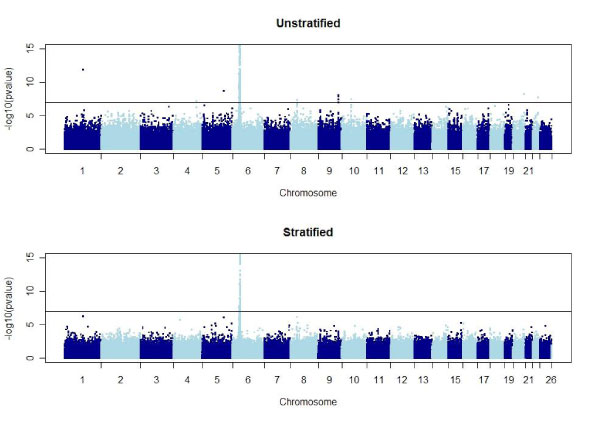
**Comparison of GWA results for unstratified, stratified analyses (5 strata)**. Horizontal line is the Bonferroni threshold for genome-wide significance at α = 0.05.

**Figure 2:**
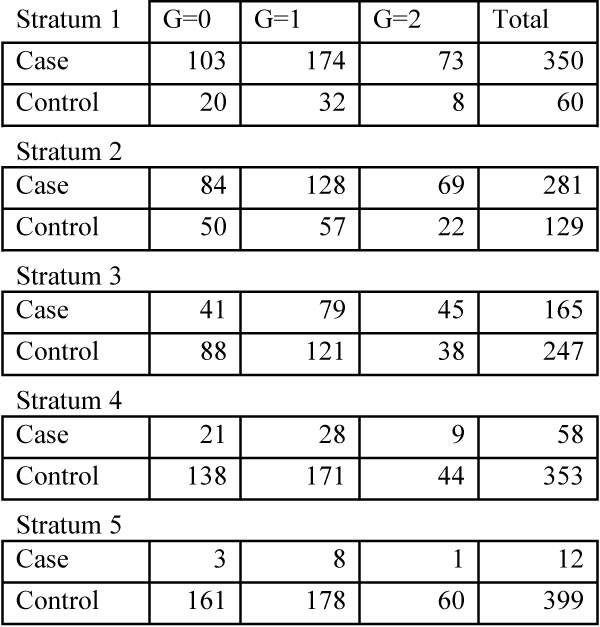
**Stratification score tables for association analysis of SNP rs3761847**.

Aside from SNPs in the HLA region on chromosome 6, genome-wide we found no SNPs that were significantly associated with RA at the α = 0.05 level (Figure 1). Interestingly, rs2900180 and rs3761847 on chromosome 9 in the *TRAF1-C5 *gene (reported by Plenge et al. [11]) and rs2476601 on chromosome 1 in the *PTPN22 *gene (reported by Begovich et al. [16]), were far from significant genome-wide (empirical adjusted *p *= 1, *p *= 1 and *p *= 0.21, respectively). To further investigate, we examined the five 2 × 3 tables for rs3761847 (Figure 2) and noted that there are only 12 cases in stratum 5. We then pooled strata 4 and 5 and recalculated the VIF to be 1.035. Pooling these strata did not increase the significance of these three SNPs (empirical adjusted *p *= 1, *p *= 1, and *p *= 0.084) and lack of statistical significance was not due to small strata size. The top three SNPs ranked by *p*-values, outside chromosome 6, were rs2476601 (chromosome 1, empirical *p*-value = 0.08), rs6596147 (chromosome 5, empirical *p*-value = 0.09), and rs1038848 (chromosome 8, empirical *p*-value = 0.21).

## Conclusion

Differences in recruitment of cases and controls suggest that control of population stratification is crucial for a proper analysis of these data. This is confirmed by the large VIF for the unadjusted analysis. Stratification score analysis dramatically reduces the VIF, increasing confidence in any associations that are found. Interestingly, once we controlled for population stratification, we found no SNPs outside the HLA region on chromosome 6 that were associated with rheumatoid arthritis at the genome-wide significance level of α = 0.05.

Like all stratified analyses, the stratification score approach will tend to lose power relative to a pooled (unadjusted) analysis when there is no confounding. Thus, our failure to replicate the associations found previously in these data may result from a loss of power from using the stratification score approach. However, the large VIF for these data makes confounding highly likely and, therefore, a competing explanation is that residual stratification in the primary analyses led to false associations. Further, Epstein et al. found that the stratification score approach had comparable power compared with other methods for control of population stratification [7]. Finally, we note that a spurious association may replicate if population stratification is not fully controlled in each analysis.

## List of abbreviations used

IBS: Identity-by-state; LD: Linkage disequilibrium; NARAC: North American Rheumatoid Arthritis Consortium; PC: Principal-component; PLS: Partial least squares; RA: Rheumatoid arthritis; SNP: Single-nucleotide polymorphism; VIF: Variance inflation factor

## Competing interests

The authors declare that they have no competing interests.

## Authors' contributions

SMS and ASA cleaned and analyzed the data. All authors participated in the design of the study and the writing of the manuscript.
